# New Sperm Morphology Analysis in Equids: Trumorph^®^ *Vs* Eosin-Nigrosin Stain

**DOI:** 10.3390/vetsci8050079

**Published:** 2021-05-06

**Authors:** Sabrina Gacem, Jaime Catalán, Iván Yánez-Ortiz, Carles Soler, Jordi Miró

**Affiliations:** 1 Equine Reproduction Service, Department of Animal Medicine and Surgery, Veterinary Faculty, Autonomous University of Barcelona, 08193 Bellaterra, Spain; swp.sabrina.gaceml@gmail.com (S.G.); jcatalan@gmail.com (J.C.); ivan.yanez22@gmail.com (I.Y.-O.); 2 Departamento de Biología Celular, Biología Funcional y Antropología Física, Universitat de València, 46100, Burjassot, Valencia, Spain. Proiser R+D, Scientific Park, University of Valencia, C/ Catedràtic Agustín Escardino, 9, Building 3 (CUE), Floor 1, 46980 Paterna, Spain

**Keywords:** horse, donkey, sperm morphology, eosin-nigrosin stain, Trumorph^®^

## Abstract

The evaluation of the male fertility potential is based on the analysis of the basic spermatic characteristics of concentration, motility and morphology. Thus, the study of sperm morphology is a fundamental element in the seminal analysis, but its real meaning has been biased by the techniques used for its evaluation. These techniques involve dehydration phases and subsequent staining, which involves the production of artifacts. The aim of the study is to compare two methods for equid semen morphology evaluation, Trumorph^®^ using living sperm vs. eosin-nigrosine stain. A total of 49 ejaculates from stallions and donkeys were used. After semen collection and dilution, an aliquot was placed on the slide and introduced in the Trumorph^®^ device. Then observation was made with a 40x objective and negative phase-contrast microscope. Another aliquot was stained using eosin-nigrosine stain and viewed using 100× magnification. Well-formed sperm were observed, and different abnormalities were identified using Trumorph^®^. The use of eosin-nigrosin staining method and Trumorph^®^ led to the same results and both techniques can be used for stallion and donkey sperm morphological analysis. However, considering the fact that Trumorph^®^ uses living sperm helps prevent sperm cell alteration during sample preparation. Therefore, Trumorph^®^ can be a good alternative to the conventional staining method, which provides a quick test on live sperm.

## 1. Introduction

The common use of spermatozoa in assisted reproduction is expanding especially for freezing or FIV, leading to a steady strengthened interest intrigued with the distinguishing sub-fertile or infertile males [1,2,3,4,5]. The evaluation of the male fertility potential is based on the analysis of basic sperm characteristics motility and morphology. Morphology data are for high predictive value for male fertility potential [6,7,8,9,10,11] and are critical for selection using the assisted reproductive techniques treatment. Moreover, despite reports on the only percentage of normal and abnormal sperm, an in-depth evaluation is necessary on the types of abnormalities present which will be important in selecting the type of clinical procedure to be adopted for intrauterine insemination, in vitro fertilization, or intracytoplasmic injection [12]. Therefore, different stains and herbal dyes have been used for morphology analysis, resulting in ambiguous outcomes [13,14,15,16]. In this regard, eosin-nigrosin staining has remained the most commonly used technique in equids thus allowing detection of morphological abnormalities and determination of viability [16]. However, it is important to mention that the staining technique passes through three steps (staining, smearing, drying), and each one critically affecting sperm morphology analysis. 

Today, with advancing technology, a new technique has been introduced for sperm morphology analysis (Trumorph) in humans and other species such as crocodile, mouse and other species [17,18]. Trumorph^®^ permits image captures of living and unstained sperm. Thus, focusing on two physiobiological points, one is the immobilization of the sperm by temperature [19], and the second is the pressure applied on the sample that leaves the sperm flattened parallel to the surface [17,19]. Further, it does not affect the viability or the morphology of the spermatozoa [14,17,19]. 

The need for a standard sperm morphological analysis is important towards establishing a technique that would enable a precise and unambiguous analysis in equid spermatozoa. Therefore, the present study was designed to compare two different methods (eosin-nigrosin staining vs wet preparation with Trumorph^®^ machine) for evaluating sperm morphology of stallion and donkey. The aim is to determine the correlation among the methods and their application to this field. We hypothesized that the Trumorph^®^ technique does not alter the various sperm parts’ shape and may provide real sperm morphology compared to staining technique in donkey and stallion.

## 2. Materials and Methods

### 2.1. Animals

The study used two ejaculates of 14 stallions and three of 7 donkeys, all collected on separate days. Animals were chosen randomly not taking into consideration their fertility condition. The equids were housed in the Equine Reproduction Service of the Autonomous University of Barcelona (Bellaterra, Cerdanyola del Vallès, Spain). This is an EU-approved semen collection center (Authorization code: ES09RS01E) that operates under strict protocols of animal welfare and health control. All animals were semen donors and were collected under CEE health conditions (free of equine arteritis, infectious anemia and contagious metritis). Since this service already runs under the approval of the Regional Government of Catalonia (Spain) and because no manipulation of the animals other than semen collection was carried out, the Ethics committee of our institution indicated that no further ethical approval was required.

### 2.2. Semen Collection

Ejaculates were collected through a Hannover artificial vagina (Minitüb GmbH, Tiefenbach, Germany) and an in-line nylon mesh filter was used to separate the gel fraction from the semen. Upon collection, gel-free semen was diluted 1:5 (v:v) with Kenney extender, previously preheated to 37 °C. The samples were incubated at 20 °C until needed for slide preparation. Once in the laboratory, various parameters of importance for seminal fertility were analyzed, such as the concentration using a haemocytometer (Neubauer chamber; Paul Marienfeld) and motility by mean of CASA-Mot system (Proiser R+D, SL, Paterna, Spain).

Sperm morphology was evaluated upon arrival of semen samples at the laboratory, each ejaculate was analyzed with the two methods, Trumorph^®^ (Proiser R+D, Paterna, Spain) system and eosin-nigrosine stain.

### 2.3. Morphological Examination of Spermatozoa

#### 2.3.1. Trumorph^®^

**Figure 1:**
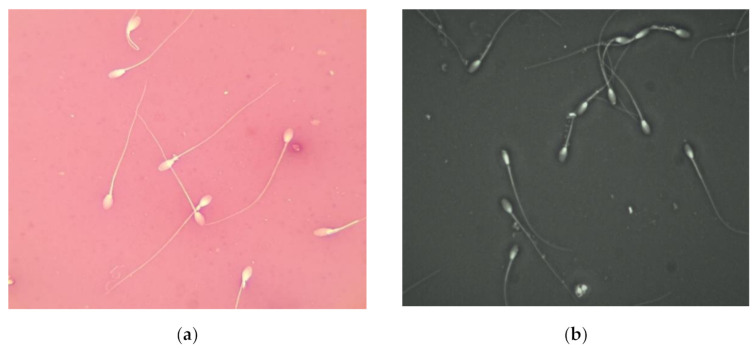
Sperm morphology of stallion. (**a**) Stallion sperm stained using eosin-nigrosin stain at ×1000 magnification; (**b**) immobilized stallion sperm using Trumorph^®^ technique at ×400 magnification.

For slide preparation, 2 and 3 μL drops of diluted semen of donkey and stallion were used, respectively. The drops were placed on the middle of the slide and covered with a 24/24 mm coverslip. Afterward, the preparation was introduced 2 times into the Trumorph^®^ system (Proiser R+D, S.L., Paterna, Spain, based) to have a total immobilization of all sperm (Figure 1). The machine contains a plate with a preheated stage predefined at a temperature of 60 °C, controlled by a dual-laser infrared thermometer (DT-8861; ATP, Leicestershire, UK), that allows the maintenance of the sample temperature at 45 °C, which is ideal for sperm immobilization. The slide is also submitted to a pressure of 6 KP, allowing the sample to extend and the sperm to flatten in an adequate volume and space.

Sperm were looked at with an optical microscope specifically designed and equipped with a 40× objective of negative phase contrast (UB203/Proiser R+D, SL, Paterna, Spain), equipped with a CCD video color camera (Proiser C13-ON, Proiser R+D) of a resolution of 768 × 576, 8/12 bits per pixel and with room temperature.

An ISAS^®^ v1 (Integrated Semen Analysis System) (Proiser R+D, S.L., Paterna, Spain) was used with the software corresponding to the morphology module to capture the microscopic fields. From each sample, a minimum number of 100 fields was captured randomly comprising around ten cells per field. For each ejaculate it was counted a minimum of 200 cells. 

#### 2.3.2. Eosin-Nigrosine Staining 

The stain preparations were made according to the following procedure: a drop of semen (5 µL) was placed on a slide preheated to 40 °C and mixed with the same volume of the dye mixture (one part 5% bluish eosin solution (Carl Roth Gmbh+Co. KG, Karlsruhe, Germany) to four parts of 10% nigrosin aqueous solution (Sigma-Aldrich, Saint Louis, MO, USA) using a glass rod to produce a smear on the slide. The samples were air-dried at room temperature. Samples were evaluated under a bright field optical microscope (Carl Zeiss, Göttingen, Germany) at ×1000 magnification using immersion oil. Three replicates were made for each ejaculate and the mean was calculated. All slides were scored blind with a minimum of 200 sperm/sample counted and classified for morpho-abnormalities.

### 2.4. Sperm Abnormalities Classification

The morphological abnormalities were counted as a percentage of the total number of counted spermatozoa. Morphological categories used in this study were: 1. Abnormal heads (including pear-shaped, narrow at the base, abnormal contour, undeveloped, lose abnormal head, narrow, big, little-normal, short-broad), 2. Loose heads (including both those with normal and abnormal head morphology), 3. Abnormal midpieces, 4. Proximal cytoplasmic droplets, 5. Distal cytoplasmic droplets, 6. Bent tail, 7. Coiled tails. 

### 2.5. Statistics

A statistical analysis of the sperm abnormalities obtained with the two seminal evaluation techniques (staining and Trumorph^®^) was performed in each animal species (horse and donkey). Shapiro–Wilk test was applied to verify the normality of the data and the Levene test to verify the homoscedasticity of the variance. When necessary, the data were transformed with arcsin √x to perform the analyzes. The difference in the means of each study variable was compared with the student T-test. Significance was set at *p* < 0.05. All calculations were performed using the Statistical Analysis System SAS 9.4.

## 3. Results

The different sperm abnormalities were well observed using the eosin-nigrosine staining technique (Figure 1a) as well when using the Trumorph^®^ technique (Figure 1b)

**Figure 2:**
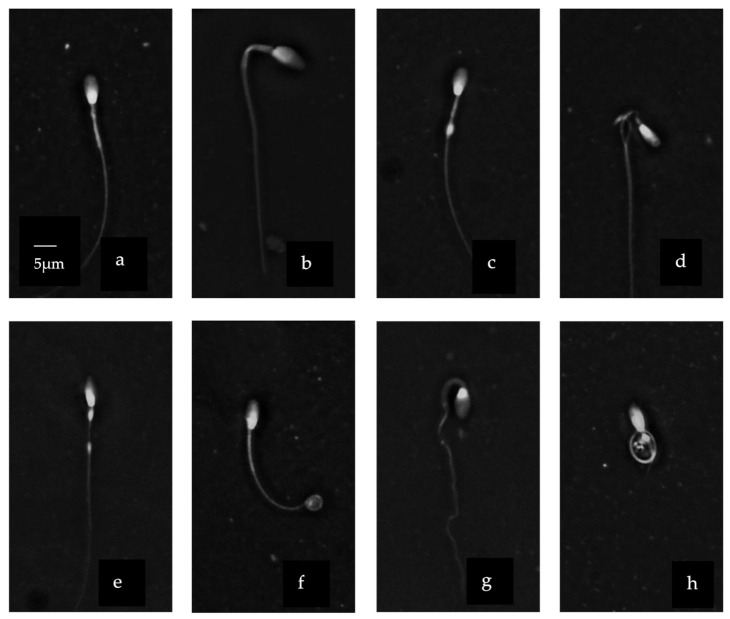

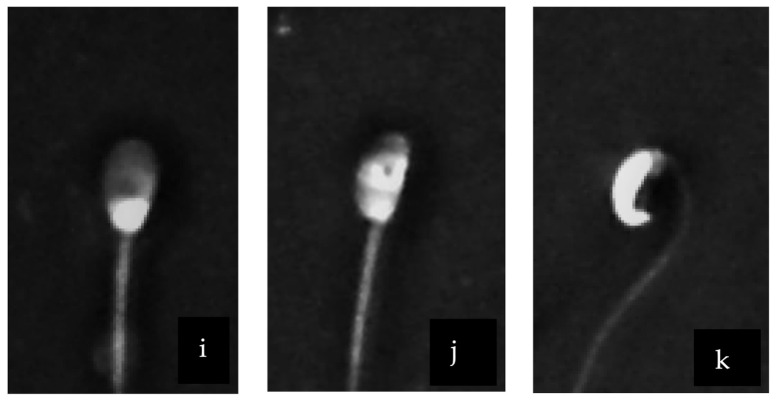
Sperm morpho-abnormalities of donkey using Trumorph^®^ technique at 400× magnification. (**a**) Roughed mid-piece; (**b**) macrocephaly with bend mid-piece; (**c**) segmental aplasia of the mitochondrial sheet in the middle; (**d**) protrusion of axonemal fiber; (**e**) distal cytoplasmic droplet (**f**) distal coiled tail with retained cytoplasm; (**g**) coiled tail with retained cytoplasm; (**h**) abnormal tail formation; (**i**) normal head with defined acrosome; (**j**) nuclear vacuole in the head; (**k**) head with cytoplasmic invagination and aplasia of mid-piece. Scale bar in (**a**) applicable to all micrographs

Stallion and donkey sperm heads were heterospermic both in size and shape (Figure 2). The head region showed a variety of different shapes and sizes. A normally shaped head (Figure 2i) was contrasted with pyriform (Figure 2k), macrocephaly (Figure 2b) or narrow (Figure 2d) shaped heads. We observed also a vacuole in the head (Figure 2j). Moreover, asymmetrical/abaxial insertion of the midpiece was detected (Figure 2c,j). The midpiece was irregular in its width in some cells (Figure 2a,b,c) and absent in others and also showed some cytoplasmic residue (Figure 2c,d). The tail showed variable degrees of curvature including some that were highly coiled (Figure 2 f,g). Stallion and donkey sperm heads were heterospermic both in size and shape (Figure 2). The head region showed a variety of different shapes and sizes. A normally shaped head (Figure 2i) was contrasted with pyriform (Figure 2k), macrocephaly (Figure 2b) or narrow (Figure 2d) shaped heads. We observed also a vacuole in the head (Figure 2j). Moreover, asymmetrical/abaxial insertion of the midpiece was detected (Figure 2c,j). The midpiece was irregular in its width in some cells (Figure 2a,b,c) and absent in others and also showed some cytoplasmic residue (Figure 2c,d). The tail showed variable degrees of curvature including some that were highly coiled (Figure 2f,g).

The morpho abnormalities count is summarized in Table 1. No significant difference was observed for various abnormalities and total abnormalities using the stained and unstained technique for stallion and donkey sperm, see Table 1.

The percentage of total abnormalities was higher in the stallion than a donkey.

## 4. Discussion

The percentage of morphologically normal and abnormal sperm are widely regarded as the most accurate and precise diagnosis of fertility, and can be correlated with diseases and exposure to reproductive toxins and hazards [6,14,20,21]. Hence, the key problem faced when evaluating of sperm morphology and morphometry is the lack of standardization with respect to the choice of staining techniques which remains greater attention in the literature. Moreover, the use of dyes with different pH, osmolarity and procedure length may affect the shape and size of spermatozoa, and thus the result of the sperm morphology evaluation. This study represents the first report comparing sperm abnormalities using a Trumorph^®^ technique and the commonly used eosin-nigrosine stain in stallion and donkey.

The use of staining for sperm morphology analysis might induce morphologic and morphometric alteration of the stallion spermatozoa [22,23,24,25]. However, a study on staining semen smears of purebred Arabian horses demonstrated that the applied staining method of eosin-nigrosine had little influence on the frequency of the observed morphological abnormalities [26]. Another study [23] found high stallion semen abnormalities using eosin-nigrosine than Papanicolaou stain. Those, it is important to note that, the presence of a high sperm defect may not always mean artifact because this can be interpreted through a more concise methodology thus revealing sperm abnormalities. Nevertheless, when studying sperm morphometry, significant differences between staining techniques in head and mid-piece sizes may be seen [24]. However, the main sources of variation in sperm morphometry are not only related to the staining technique. Effectively, many factors such as the sample preparation, fixation method, microscopic system (optics and camera), and the activity of the technician can influence the repeatability of the analysis and its comparison among laboratories [23].

According to guidelines by the Society for Theriogenology (SFT), the evaluation of stallion sperm morphology should be performed on fresh unstained samples using a microscope with phase contrast [7]. However, there are very few technicians skilled in this technique in most andrology and veterinary laboratories as well as the scarce resources due to the financial implication. This is why we chose the Trumorph^®^ technique which was previously studied, validated and tested [17]. Our results herein showed that the Trumorph^®^ technique permitted to have complete sperm shape observation with a clear boundary of its head, midpiece and tail. It permitted one to see the multiple abnormalities that are observed using eosin-nigrosin stain. Both methods were found to be acceptable for morphological evaluation of fresh stallion and donkey spermatozoa. 

Trumorph^®^ technique keeps the sperm alive and immobile, without the use of fixing agents that damage them. It should be noted that in previous work carried out on sperm of various species of mammals, the maintenance of cell life could not be assured [19]. The restriction of cell mobility is a consequence of the 45 ° C thermal shock provided by the system stage [27]. Recently, various works have been carried out with semen of carp and eel, which has proven that the use of Trumorph^®^ does not damage cell life. In the case of carp, the application of pressure by the system led to the activation of sperm motility without the need to suspend the cells in the activation medium [28]. It has been proposed that this is a consequence of the cells being subjected to a natural pressure in spawn conditions at a certain depth. Regarding the eel, it was observed that for at least eight minutes after the application of the pressure, the cells varied their area (and therefore their volume) with oscillating values, which indicates a response by a full and fully functional membrane [28]. 

In the present study, the comparison between the efficiency of the two methods (live vs dead sperm), it was found that despite the sperm being alive or dead, the incidence of sperm morphology defect remains the same. An explanation to our result could be as a result of a fresh semen used for the preparation of eosin-nigrosin stain. In this regard, previous studies have demonstrated the effect of cooling on membrane permeability [29,30]. Therefore, the percentage of abnormalities increases when using different stains on cooled semen due to the effect of different staining techniques. Effective cooling significantly alters sperm lipid fraction, increase membrane permeability, reduce enzyme activities, change membrane proteins [31] and causes cholesterol loss [32] with concomitant reduction of the stability of sperm cell membrane [33]. However, the lack of differences observed between both methods in our study may be due to the fact that fresh semen was used, therefore further studies are needed on cooled or frozen-thawed semen using Trumorph^®^ for a clearer understanding but also a study of sperm morphometry will enlarge our understanding of its real effect on sperm.

In practice, however, both techniques offer different possibilities. The eosin-nigrosin is a negative differential dye which identifies intact live and dead sperm. However, this technique needs an experienced technician for smears and sperm identifications. Using Trumorph^®^ all the sperm have a white color (living and dead sperm). Nevertheless, the method is simple since it includes one step preparation, it’s fast, and easy to learn and perform consistently by everyone and this is a primary criterion for robustness in results. The observations are made at 400× magnification by taking images that are observed by a computer which can be easily registered. In this regard, the last method diminishes the abnormalities that may be caused by the staining and preparation processes as observed when eosin-nigrosin is used. Those resulting in better and correct interpretation of abnormalities results when using Trumorph technique, then accurate male fertility assessment. However, fertility is not only related to morphology those more analysis are needed.

Moreover, if we compare both techniques’ cost, we find that Trumorph^®^ can be a profitable method compared to the staining technique. Trumorph^®^ technique needs a low-cost machine and a microscope to catch sperm images while eosin -nigrosin staining needs a certain preparation of chemical dye continuously and can be costly. Further, it is important to note that the chemical dye used to stain the sperm is not biodegradable and hazardous to the environment. Many studies using natural die were performed recently as a way to replace the chemical dye used in sperm morphology assessment [15]. 

## 5. Conclusions

Obtained observations lead to the conclusion that it is very important to establish the natural dimensions of the unstained sperm, and only then we can determine the optimal technique and the reference values for this technique. Moreover, it is important to select the right technique for sperm morphology assessment that permit an easy, fast, and reliable results. For that, Trumorph^®^ could be a good alternative for a right morphology classification and diagnostic of fertility disorders. 

## Figures and Tables

**Table 1:** Morphological abnormalities of sperm in relation to the technique used (means ± SD).

·	Stallion			Donkey		
Variables (%)	Stain	Trumorph^®^	*p*-Value	Stain	Trumorph^®^	*p*-Value
Total abnormalities	27.72 ± 12.74	29.78 ± 10.13	0.29	15.40 ± 11.37	15.11 ± 10.56	0.12
Middle piece	2.83 ± 2.67	3.05 ± 3.03	0.48	0.63 ± 0.60	0.32 ± 0.35	0.85
Proximal droplet	5.56 ± 6.80	7.55 ± 5.00	0.94	0.96 ± 1.33	0.88 ± 0.98	0.06
Distal droplet	2.70 ± 4.17	1.90 ± 3.90	0.52	0.33 ± 0.59	0.94 ± 1.05	0.95
Bent tail	7.48 ± 7.87	9.69 ± 7.16	0.30	4.35 ± 6.44	8.90 ± 9.04	0.09
Coiled tail	3.66 ± 3.09	2.70 ± 3.47	0.69	6.85 ± 11.38	1.42 ± 3.33	0.92
Abnormal head	2.55 ± 2.35	2.79 ± 1.80	0.78	1.28 ± 1.29	1.33 ± 1.08	0.50
Loose head	2.94 ± 3.60	2.10 ± 2.79	0.24	1.00 ± 1.17	1.32 ± 1.41	0.10

SD: standard deviation, stain: eosin-nigrosin, significant difference if (*p* < 0.05).

## Data Availability

The data that support the findings of this study are available from the corresponding author J. M, upon reasonable request.
